# The Influence of the Machining Parameters of AW-7020 Aluminum Alloy Shafts on the Surface Roughness, Cutting Forces, and Acoustic Emission Signal

**DOI:** 10.3390/ma18091992

**Published:** 2025-04-28

**Authors:** Krzysztof Dudzik, Wojciech Labuda

**Affiliations:** Faculty of Marine Engineering, Gdynia Maritime University, 81-225 Gdynia, Poland; w.labuda@wm.umg.edu.pl

**Keywords:** acoustic emission (AE), cutting forces, turning, cutting parameters, surface roughness, diagnostic, monitoring, AW-7020 aluminum alloy

## Abstract

To ensure high quality of the machined surface, various methods are used to assess the turning process. This process can be monitored using indirect techniques, such as measuring cutting forces and recording acoustic emission (AE) signals, which help determine the stability of machining conditions. The tests were carried out on AW-7020 alloy shafts turned using a tool with a replaceable CCGT09T302-DL insert. Cutting forces were measured using a Kistler dynamometer, while AE signals were recorded with a system from Physical Acoustics Corporation. Surface quality was evaluated based on roughness measurements, with the Ra parameter ranging from 1.67 to 5.03 μm. An increase in cutting forces, particularly the Fz component, resulted in higher surface roughness. The Fz force ranged from 41 to 251.8 N. Parameters of the AE signal made it possible to identify the most stable turning conditions. For this purpose, the standard deviation of the selected parameters—such as amplitude and RMS—was compared. Additionally, spectral analysis of the AE signal allowed observation of frequency-related changes. The test results indicated that the most stable cutting conditions—and, consequently, the best surface quality—were achieved for the sample machined with the following parameters: Vc = 300 m/min, ap = 0.5 mm, f = 0.078 mm/rev.

## 1. Introduction

The process of turning machine parts is still relevant, which is why many research works on this subject appear both in industry and in research and scientific institutions. These are mostly used in the automobile, gear, die, and bearing industries [1]. During the turning process, many factors affect the final dimensions and quality of the machined surface. The input factors that determine the process carried out on the machine tool include basic cutting parameters. These include cutting speed, feed, and cutting depth. The constant factors that have a direct impact on the final quality of the detail include, among others, the type of the machine tool, the material of the turning tool and its geometry, the material of the workpiece, and the type of the cooling and lubricating liquid. In the turning process, there are also disturbing factors that manifest themselves through vibrations in the system (machine tool, holder, workpiece, tool), wear processes of the cutting edge, or inhomogeneity of the workpiece. The output factors of the finishing turning process are primarily parameters of the geometric structure of the surface. The abovementioned factors may cause the use of unchanged technological parameters of machining to result in different measured values of the geometric structure parameters of the shaft surface.

In various research centers, researchers deal with the mentioned issues. Monitoring of machining processes is an important issue, because it allows control over the process of cutting tool wear, and thus over the quality of manufactured parts. Feng et al. [2] and Kuntoğlu et al. [3] presented a comparison of the usefulness of various methods of monitoring the machining process, including tool wear. They presented the results of tests of vibration, acoustic emission, cutting forces, temperature, lathe drive power, etc. Asiltürk et al. [4] additionally analyzed the surface roughness of machined AISI 4140 steel at various turning parameters, correlating it with the diagnostic parameters: vibration and acoustic emission. Maia et al. [5] also used the relationship between the parameters of the acoustic emission signal and surface roughness, proposing real-time monitoring of the tool condition. They indicated various methods of data processing, including STFT (short-time Fourier transform) and FFT (fast Fourier transform). Bhuiyan et al. [6] presented the possibility of monitoring the condition of the tool during its operation as well as the deformation of the machined material and the chips formed at that time.

Dudzik et al. [7] presented the possibility of applying acoustic emission and dynamometric methods for monitoring the turning process of stainless steel 304L. Both the recording of forces during turning and the RMS (root mean square) of the AE signal allowed the determination of individual stages of the cutting tool insert wear. Monitoring of tool wear during hard turning of the Inconel 718 material, also using acoustic emission, was presented in the research work of Rao et al. [8]. Similarly, Bhuiyan et al., in their research, used the phenomenon of acoustic emission and, additionally, the vibration signature to monitor tool wear and chip formation and analyze the surface roughness of the workpiece under various cutting conditions [9].

A multi-sensor system for monitoring the cutting process in terms of catastrophic tool failure (CTF) was presented in the publication by Balsamo et al. [10]. The authors used a system consisting of a force sensor and acoustic emission. Material removal during high-efficiency cutting should be controlled. First of all, it is necessary to ensure the correct shape and size of the chip. Therefore, one of the most important factors that should be considered when machining various materials, especially difficult-to-cut materials, is the proper selection of technological process parameters. It should guarantee low cutting forces, reduced tool wear, correct chip fragility, and the required quality of the machined surface [11]. Turning difficult-to-cut materials is an important aspect of research conducted along with their development, simultaneously with new tools for their processing. An example of research on such materials is the article by Schmidt et al., who used, among others, M42 tool steel, tungsten tantalum alloy, aluminum 6061, and Nitronic 33 stainless steel [12].

Aluminum alloys require strictly specified cutting conditions during their machining process because their machinability differs from that of other materials. Due to the problems occurring during machining, they can be classified as difficult-to-machine materials. This is due to the properties of aluminum alloys, such as the high coefficient of linear expansion (for steel, this factor is two times lower) and high thermal conductivity. Many researchers deal with the issues of machining various types of aluminum alloys, which are machined under variable conditions. Nataraj et al. researched the influence of turning process parameters on the machinability of a hybrid metal matrix composite containing alumina (Al_2_O_3_) and molybdenum disulfide (MoS_2_) particulates dispersed on aluminum casting alloy LM6 in the turning process [11]. Aluminum alloy 6082 was used in studies related to the optimization of parameters in the CNC turning process by Palaniappan et al. [13]. Other researchers (Karthikeyan et al.) investigated the 6063 aluminum alloy, in which the optimization of CNC turning parameters was performed using an experiment based on the Taguchi method [14]. Another example of research using aluminum alloys is the article by Otieno et al., who investigated the machinability of rapidly solidified aluminum [15]. In scientific journals, researchers present tests based on cutting process simulations, in which different software tools were used. The accuracy of any software tool can be guaranteed only by providing accurate material properties concerning material constants. Issues related to the results of cutting force simulations obtained from Deform-3D finite element software during dry turning of the 7075 aluminum alloy, along with the experimental results, were reported by Mali et al. [1]. Teimouri et al. presented experimental studies and analysis of machining parameters during the turning of the 7075 aluminum alloy, along with the development of an empirical model [16]. Pan et al. presented FEM simulations of the damaged area with material separation at the nose radius of the tool [17]. In an article by Saravanan et al., the 6082 aluminum alloy blended with a boron carbide-reinforced material by mechanical mixing was examined [18]. The researchers performed SEM analysis, and the castings were machined on CNC machines. Machinability studies were compared with the mono-alloy and composite materials. Therefore, it is important to conduct experimental tests to learn about the required material constants and the effect of machining conditions on the cutting process. An appropriately rich database obtained from the studies can be the basis for optimizing machining and forecasting both the state of tool wear and ensuring the required surface quality using modern methods, such as machine learning, deep learning, AI, etc. Krishnakumar et al., in their study [19], presented a tool condition monitoring system based on advanced signal processing techniques and artificial intelligence models. The condition of the tool was assessed using acoustic emission and vibration signals recorded during the machining of the Ti–6Al–4V titanium alloy. The article [20] by Shah et al. focused on machine learning methods, including deep learning, based on laboratory tests conducted during the milling of cast iron with simultaneous recording of acoustic emission signals. The study aimed to monitor tool wear and predict catastrophic failure. The results can serve as a foundation for creating a comprehensive database and implementing SHM methods in industrial applications. Trends in SHM systems for monitoring tool condition, driven by increasing requirements for the quality of machined surfaces and the new possibilities offered by technological advancements, were presented by Tran et al. [21]. The study highlights the potential of modern computer systems and advanced data processing methods in the context of Industry 4.0, such as artificial intelligence (AI), deep learning (DL), the Internet of Things (IoT), and the Industrial Internet of Things (IIoT). Signals from various systems were recorded, including cutting forces, acoustic emission (AE), vibrations, and others.

The tests were performed using shafts made of AW-7020 aluminum alloy. They exhibit higher strength properties than the most commonly used alloys 5xxx (Al–Mg) and 6xxx (Al–Mg–Si). The disadvantage of Al–Zn–Mg alloys is their susceptibility to stress corrosion and lamellar corrosion, which limits their application [22]. Al–Zn–Mg alloys exhibit good machining ability and dimensional stability. Further, 7xxx alloys are widely used in the production of various machine parts, motor vehicles, rolling stock, as well as structural elements of ships and aircraft [23]. The determination of cutting forces during turning processes allows for the assessment of the material’s machinability. The extent of cutting forces is related to the amount of heat in the cutting area, tool wear, surface quality, and accuracy of the workpiece. It is assumed that aluminum alloys have better machinability than steel, so the force required for machining aluminum is about 30% of the force required for machining steel [24]. A commonly used alloy in this group is AW-7020. Due to its high strength and low density, this alloy has found wide application in engineering, especially where a lightweight yet durable structural material is required for manufacturing precision components. It is used in the transportation, military, aerospace, and space industries, as well as in pressure components, bicycle and motorcycle frames, and heavily loaded machine parts. It can serve as an effective substitute for steel when reduced structural weight and increased corrosion resistance are desired. During the process of turning aluminum alloys, the problem may be the correct removal of chips. The basic technological problems occurring in the process of machining aluminum alloys include removing chips from the cutting zone, the formation of adhesion to the cutting tool, the formation of surface irregularities, the wear of the cutting tool, temperature, and forces. In addition, obtaining a good quality surface of the machined shaft may be unsatisfactory due to the tendency to form a ribbon chip, which cannot be fully controlled.

Due to the great possibilities of tool configuration, machining conditions during the turning process, and other external factors interfering with the machining, it is important to experimentally measure cutting forces to determine the most favorable cutting conditions. The article presents the research results on the effect of variable turning parameters on the values of forces and monitoring the cutting process of the AW-7020 aluminum alloy using dynamometric and acoustic emission methods. The basic parameters of surface roughness and material ratio were selected to assess the surface quality of the shafts.

## 2. Materials and Methods

The research was performed on a CU500MRD universal lathe (ZMM Sliven, Sliven, Bulgaria). Shafts with a diameter of 60 mm made of aluminum alloy EN AW-7020 T6 (tempered) (WMH GROUP GERMANY, Essen, Germany) with a hardness of 100 HV were subjected to a finishing turning process. The chemical composition of the investigated alloy was taken from the manufacturer’s melt analysis and is presented in Table 1.

For machining the shafts, a tool with a replaceable cutting insert CCGT09T302-DL (Duracarb, Tieshan, China) was used. Basic information about the cutting insert: C—rhombic insert shape with point angle 80°; C—insert clearance angle 7°; G—tolerance (nose height, ±0.025 mm; thickness, ±0.13 mm; inscribed circle, ±0.025 mm); T—insert type (single-sided CB screw hole); 09—insert size = cutting edge length—9.525 mm; T3—insert thickness—3.97 mm; 02—nose radius—0.2 mm; DL—chip breaker.

To determine the effect of variable cutting conditions, the values of individual cutting parameters were selected as follows:

– cutting speed—Vc = 150; 225; 300 m/min;
– depth of cut—ap = 0.5; 1.0; 1.5 mm;
– feed—f = 0.078; 0.168; 0.236 mm/rev.

**Figure 1:**
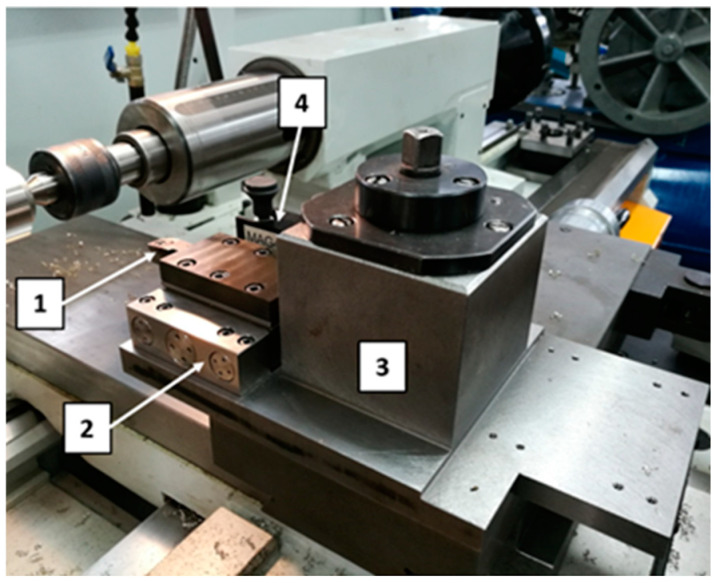
The view of the equipment used in the study: 1—tool with a removable insert, 2—dynamometer, 3—dynamometer grip, 4—acoustic emission sensor with a magnetic holder.

The view of the equipment used in the study is presented in Figure 1. The tool was mounted in a Kistler dynamometer (Kistler Instrumente AG, Winterthur, Switzerland), which enabled the measurement of cutting forces during the turning process. The setup consisted of a 9119AA2 piezoelectric dynamometer (Kistler Instrumente AG, Winterthur, Switzerland), a 5070 charge amplifier, and a computer equipped with DynoWare software (DynoWare type 2825D-02, Version 2.6.5.1.6, Kistler Group, Winterthur, Switzerland) for data acquisition and analysis. This dynamometer allows for the measurement of both dynamic and quasistatic forces: Fx—radial force, Fy—feed force, and Fz—cutting force. The range of measured forces is ± 4 kN. The equipment measures the active force regardless of the application point.

**Figure 2:**
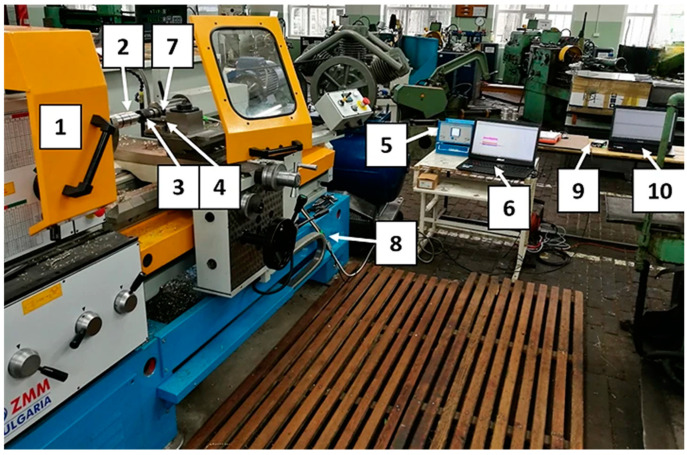
General view of the laboratory stand: 1—lathing machine, 2—shaft, 3—tool, 4—dynamometer, 5—dynamometer recorder, 6—dynamometer computer, 7—AE sensor, 8—preamplifier, 9—AE recorder, 10—AE computer.

**Figure 3:**
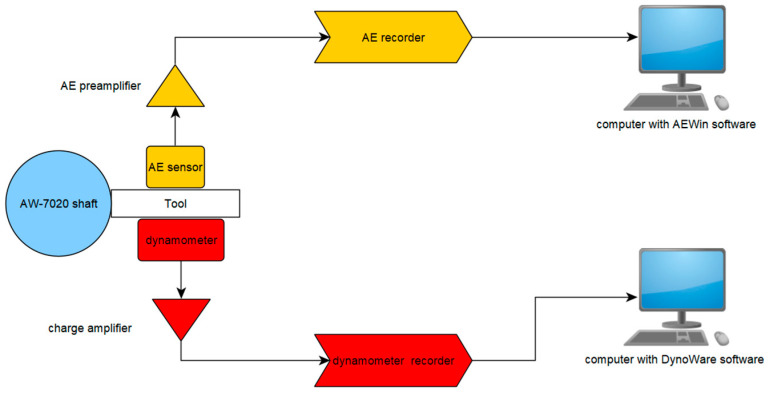
Diagram of the measuring stand.

AE (acoustic emission) research was performed using a set consisting of a single-channel recorder USB AE Node, type 1283 with bandpass 20 kHz–1 MHz, a preamplifier with bandpass 75 kHz–1.1 MHz, an AE-Sensor VS 150M (with a frequency range of 100–450 kHz), and a computer with AEWin for USB Version E5.30 software (Physical Acoustics Corporation, Princeton, NJ, USA) to record and analyze AE data. The sensor was fixed to the surface of the dynamometer using a MAG4M (Vallen Systeme, Icking, Germany) magnetic holder designed for that sensor. Silicone grease was used between the sensor and the dynamometer surface as a coupling fluid. The view of the test stand used in the study is shown in Figure 2, and its schematic diagram is presented in Figure 3.

For determinate statistical plans whose input factors (n number) take values on three levels, a three-level plan should be used. To determine the effect of variable cutting parameters on the quality of the machined surface while simultaneously monitoring acoustic emission parameters and forces occurring during the turning process, a three-level complete statistical plan was used. These plans are presented with the symbol SP/DC 3^n^, which denotes a statistical plan (SP), determinate, complete (DC) method with three levels of variability and n input factors. The total number of experiments is equal to the number of all possible combinations of input factors on three levels of variability and is 3. In practice, the SP/DC 3^3^ plan is used with the number of factors *n* = 3, for which the total number of experiments would be *n* = 27. However, Hartley experimental plans [25] are more convenient and useful for carrying out research in the field of machine technology. These plans are characterized by the so-called contrast defining, 1 = x_k_x_l_x_q_… or −1 = x_k_x_l_x_q_…, which determines the fractional part of a given plan. Hartley plans consist of three blocks of experiments, which include:

– fractional repetition of the SP/DC 2^n^ type experiment (statistical plan, determinate, complete, type two to the nth),
– experiments at star points with arm α = 1,
– experiment at the central point of the plan.

To minimize the number of experiments, a hypercube matrix design was used with a contrast defined by 1 = x_1_x_2_x_3_. The design of the research plan uses three values of each quantity under study. The maximum value is marked as “+1,” the middle value as “0,” and the minimum as “−1”. The Hartley experimental design for three input variables at three levels is widely used by many researchers in various experimental studies. Niedźwiedź et al. [26] applied this design to investigate the influence of plasma electrolytic oxidation parameters (peak current density, process duration, and pulse frequency) on the tribological properties and wettability of the produced coatings. Another article employing the Hartley design focused on the impact of variable production process parameters on the micromechanical and scratch resistance properties of oxide coatings [27]. Kluz et al. [28] presented the results of research on the influence of slide burnishing on the surface roughness of shafts made from 42CrMo4 steel. The experiment was based on the Hartley design, which enables the definition of a regression equation in the form of a second-degree polynomial. The input parameters used in the experiment included three variables: applied pressure, burnishing speed, and feed rate.

Table 2 presents the coded and real factors under study (independent variables).

In the surface roughness measurements, the directionality was assumed along the turning axis so that the tested surface corresponded to the maximum values of the analyzed parameters, and the measurement direction was perpendicular to the direction of the irregularities.

**Figure 4:**
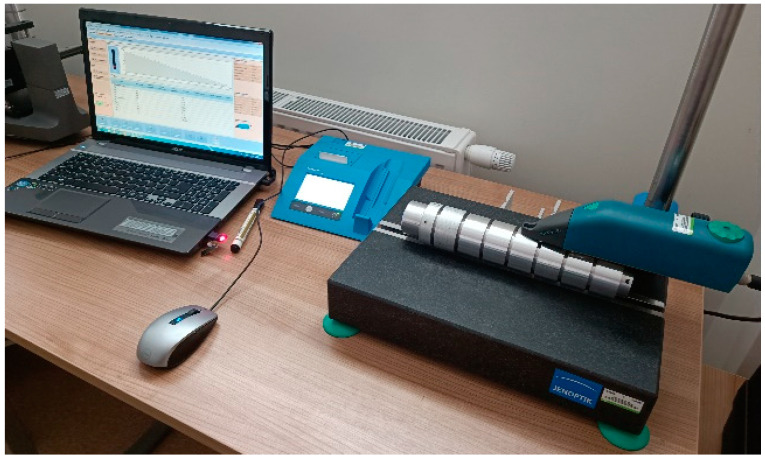
View of a surface roughness measurement.

The selected surface roughness parameters and the material ratio were measured using a Hommel-Etamic W20 profilometer (JENOPTIK Industrial Metrology Germany GmbH, Tönisvorst, Germany) (Figure 4). The measurement of the analyzed parameters was carried out for the elementary section lr = 0.8 mm and the measurement section ln = 4.0 mm, with the mapping section set to 4.8 mm. During the tests, a measurement speed of Vt = 0.5 mm/s was used. The following parameters were used to determine the effect of the machining process on the change in the height of surface irregularities:

– Ra [μm]—arithmetic average roughness;
– Rt [μm]—total height of the profile;
– Rk [μm]—core roughness depth;
– Rpk [μm]—reduced peak height;
– Rpv [μm]—reduced valley depth.

## 3. Results and Discussion

The surface quality after the machining can be determined using surface roughness parameters and the material ratio. The basic parameter used for this purpose is Ra. The applied variable cutting parameters resulted in obtaining their different measurement values. The lowest value of Ra = 1.67 µm was obtained for the shaft pin which was processed with the lowest feed and cutting depth values (f = 0.08 mm/rev, ap = 0.5 mm) and the highest cutting speed of 300 m/min. The average value of the parameter Ra = 1.68 µm, but with a much less stable process, was obtained when Vc was reduced to 150 m/min. A two-fold reduction in the cutting speed resulted in a greater scatter of results, as evidenced by the obtained values of standard deviation (0.13 µm) and standard error (0.07 µm). The turning process carried out with an increased feed and depth of cut values affected the deterioration of surface quality in the entire range of the analyzed cutting speed. The surface with the highest average value of Ra = 5.03 µm was obtained for sample No. 9. The obtained values of the Ra parameter reached in the test are shown in Table 3.

**Figure 5:**
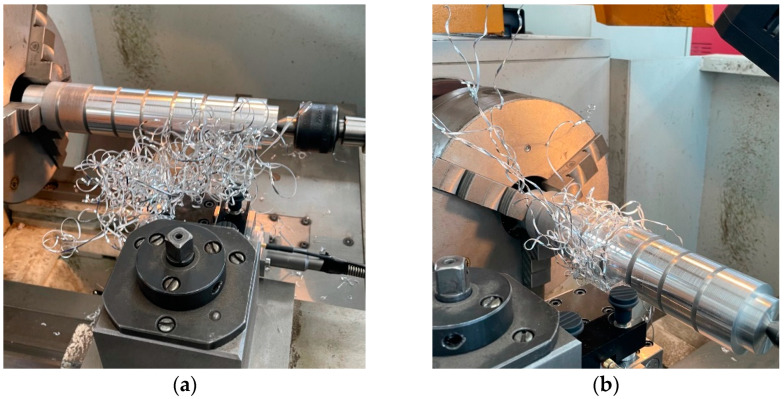
View of the cutting zone where the ribbon chip was formed: (**a**) sample No. 4, (**b**) sample No. 9.

The use of variable cutting parameters for the process of turning shafts made of EN AW-7020 aluminum alloy has a significant impact on the chip formation process. This affects the distribution of forces and often affects the quality of the machined surface. A ribbon chip can get stuck between the cutting edge and the machined surface, causing an increase in the cutting, feed, and passive forces, and thus causing scratches on the machined surface of the workpiece. Figure 5 shows situations where an undesirable chip was created in the cutting zone, which scratched the machined surface during the process. The presented photos show a situation in which the chip that was created was held directly by the cutting tool and the machined material, and when it was wound around the machined workpiece. In both cases, this affected the forces of the cutting process; this will be presented later in the article.

To more accurately illustrate the influence of variable parameters of the turning process on the quality of the machined surface, Table 4 presents the Rt parameter, and Table 5, Table 6 and Table 7 present the material ratio parameters. The lowest Rt values were obtained for the turning process with the smallest cutting depth and feed at the highest cutting speed (sample No. 2). Similar values were obtained for sample No. 1. The highest total height of the profile values were observed for samples Nos. 4 and 9. Basic statistical analysis of the obtained results of the Ra and Rt parameters also allows for determining the shaft machining conditions. Higher values of the standard deviation and standard error indicate unstable machining conditions or the formation of a ribbon chip, which can damage the machined surface.

Material ratio parameters Rk, Rvk, and Rpk provide information on the load capacity, peaks, and valleys of the surface profile, respectively. The lowest average values of the analyzed material ratio parameters were also obtained for the shaft surfaces after the turning process with the lowest feed and depth of cut values (samples Nos. 1 and 2). The highest values were obtained for the shaft surfaces processed with the highest feed (samples Nos. 4 and 9). The highest values of Rpk were obtained on the surface of shaft pins Nos. 3 and 5, where a reduced cutting speed value was used.

**Figure 6:**
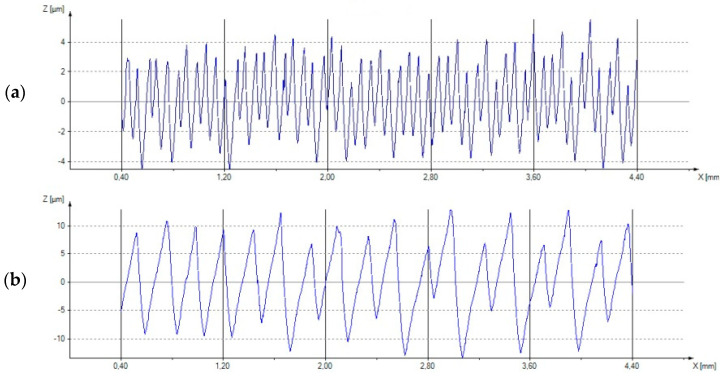
Examples of profilometers for the surfaces with (**a**) the lowest (sample No. 2) and (**b**) the highest (sample No. 4) values of the Ra and Rt parameters.

An example of a profilometer measurement for the lowest roughness value (sample No. 2) is shown in Figure 6a, while the highest roughness value (sample No. 4) is presented in Figure 6b. There were no significant scratches or surface defects on the tested measuring sections because the distribution of the recorded peaks did not deviate significantly beyond the range of the measurement peaks. The differences in the average values resulted mainly from the cutting parameters used during turning. The research showed that the feed has the greatest effect on the obtained surface roughness values.

During the implementation of the research plan, it was observed that the most favorable conditions in terms of force distribution were obtained on sample No. 2, which was subjected to machining at the highest cutting speed of 300 m/min, the lowest feed f = 0.078 mm/rev, and cutting depth ap = 0.5 mm. More than six times higher values of the Fz force were recorded for the highest cutting speed (Vc = 300 m/min), combined with the highest feed rate (f = 0.236 mm/rev) and depth of cut (ap = 1.5 mm). The results of the cutting forces recorded during the test are presented in Table 8.

**Figure 7:**
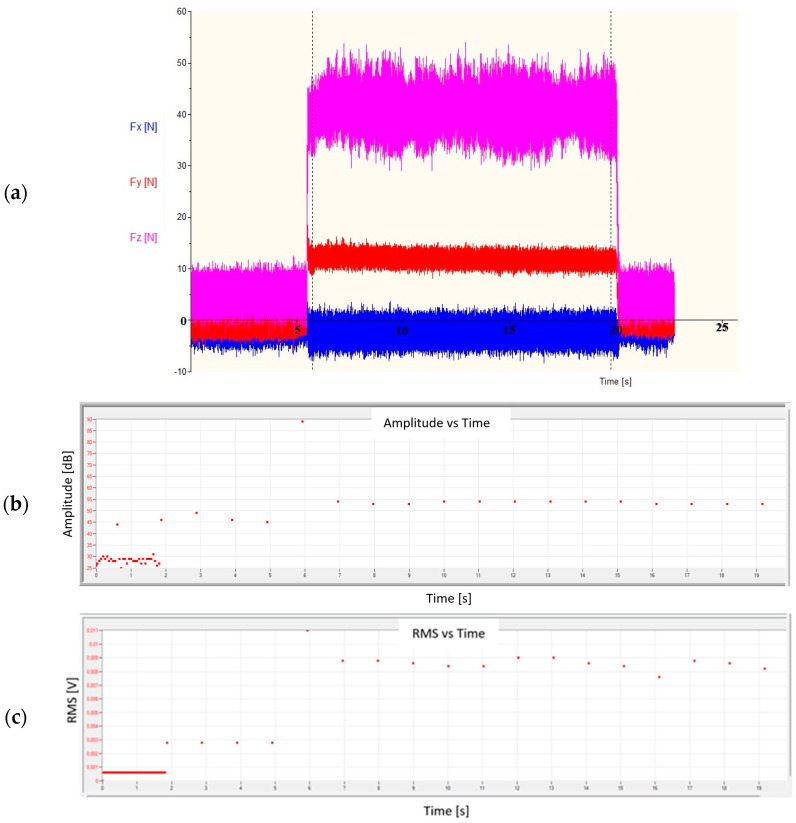
View of (**a**) forces and (**b**,**c**) the AE parameters during the turning process for stable conditions for sample No. 2.

Figure 7a shows the distribution of forces, and Figure 7b,c shows the recorded acoustic emission signal (in the form of signal amplitude and RMS) during the turning process for the most favorable machining conditions. When machining a shaft with a low value of the ap and f parameters and the highest value of Vc, stable machining conditions occurred, so the values of the recorded forces were generated continuously without any disruptions to their course. A similar relationship could be observed for changes in the amplitude and RMS of the AE signal. At the moment of contact of the cutting edge with the machined material, the values of both forces and AE parameters increased. If there were no disruptions, their values did not differ significantly from the average value.

**Figure 8:**
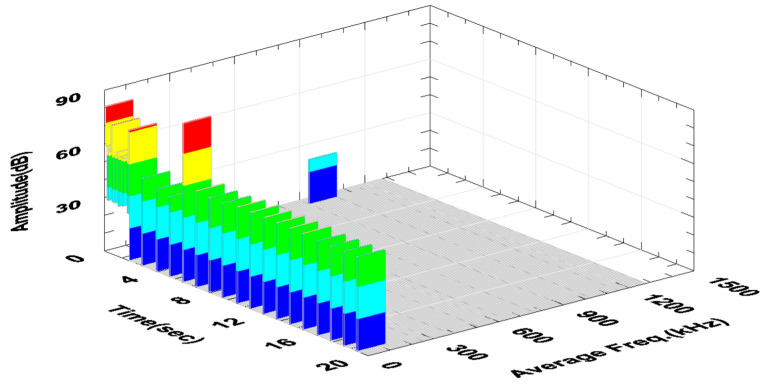
Change in the amplitude and the mean frequency of the AE signal as a function of time—sample No. 2 (software-generated colors to improve visibility of amplitude changes every 20% of the range).

**Figure 9:**
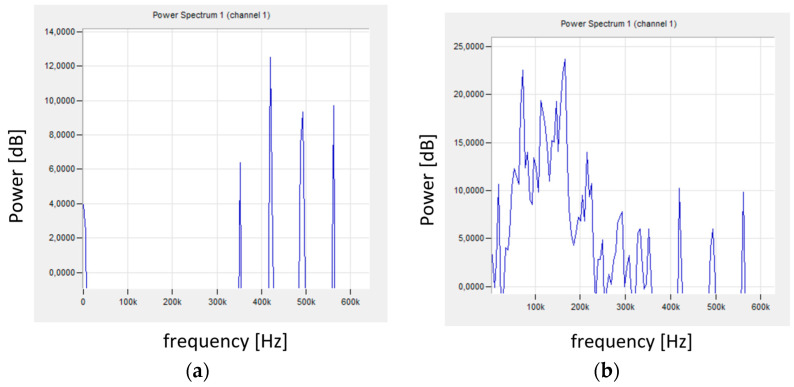
AE signal after FFT (**a**) for the initial test phase, (**b**) for stable machining conditions—sample No. 2.

The constant values of the AE signal amplitude visible in Figure 8 are characteristic of stable turning conditions. The high value of the signal amplitude in the 6^th^ s of the AE signal recording probably came from the chip breakage that entered between the tool and the workpiece. The signals with higher amplitude visible in the first phase of the recording were characterized by higher frequency and were not considered in further analyses. These signals were characterized by very low energy and were considered acoustic noise. Their spectrum is shown in Figure 9a and was compared with the signal spectrum during stable turning conditions (Figure 9b).

FFT analysis of the recorded AE signal allowed obtaining the signal spectrum and its characteristic frequencies. Figure 9a shows the signal in the initial phase of the test, when there was no contact between the tool and the workpiece. Single signals with a relatively high frequency (350–570 kHz) and very low energy could be observed. Figure 9b shows the signal spectrum during the turning process. The signal with a much higher energy has a frequency in the range of 0–560 kHz, with the most important signals with a frequency not exceeding 180 kHz.

**Figure 10:**
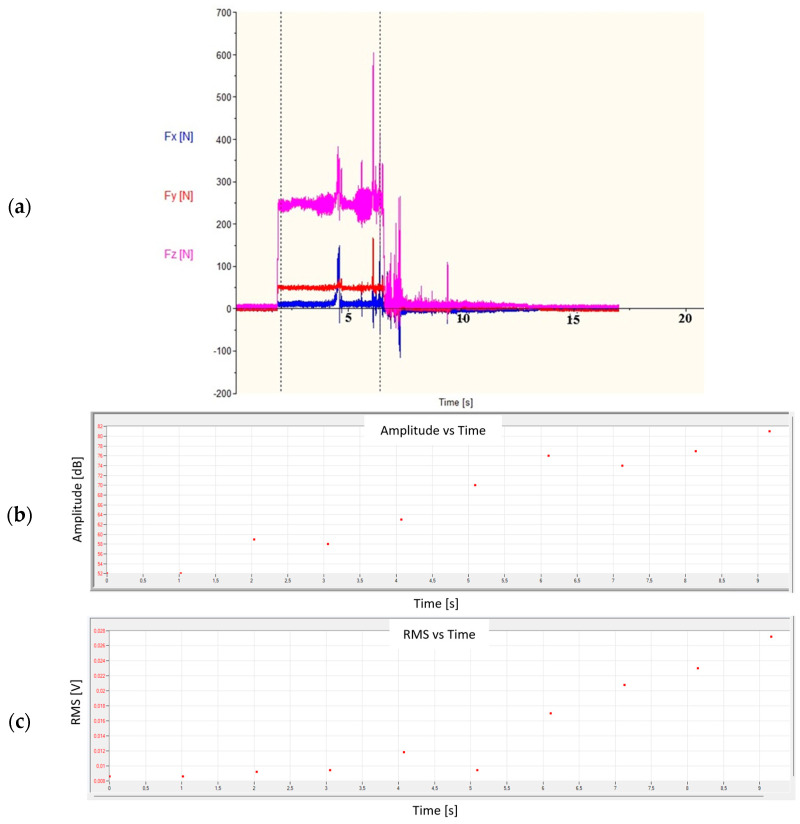
View of (**a**) forces and (**b**,**c**) the AE parameters during the turning process for unstable conditions recorded for sample No. 4.

Figure 10 shows the graphs of the change in forces (Figure 10a) and the amplitude and RMS of the AE signal (Figure 10b,c) for the cutting conditions that resulted in the formation of a ribbon chip. In the event of a disruption of the turning process, e.g., when the chip formed in the cutting zone and was not properly removed, an uncontrolled jump of the analyzed values of both forces and AE signal parameters occurred.

**Figure 11:**
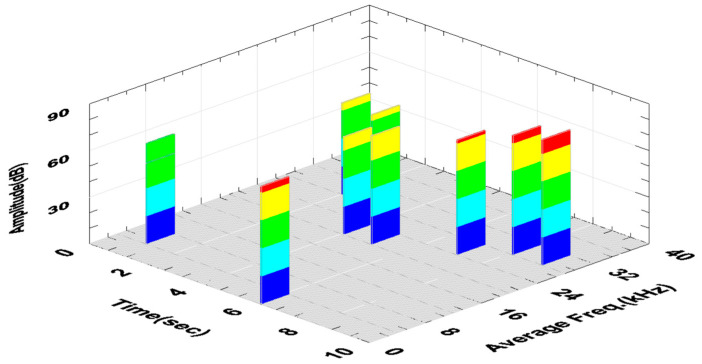
Change in the amplitude and the mean frequency of the AE signal as a function of time—sample No. 4 (software-generated colors to improve visibility of amplitude changes every 20% of the range).

The increase in the Fz force and the amplitude and RMS of the AE signal recorded in the 4^th^ second of the test indicated the formation of a ribbon chip that entered between the tool and the machined surface. This is particularly visible in the graph of changes in the RMS signal as a function of time, where this value constantly increases (Figure 10c). Figure 11 shows the changes in the amplitude and average frequency of the AE signal over time, recorded during the turning of sample No. 4.

**Figure 12:**
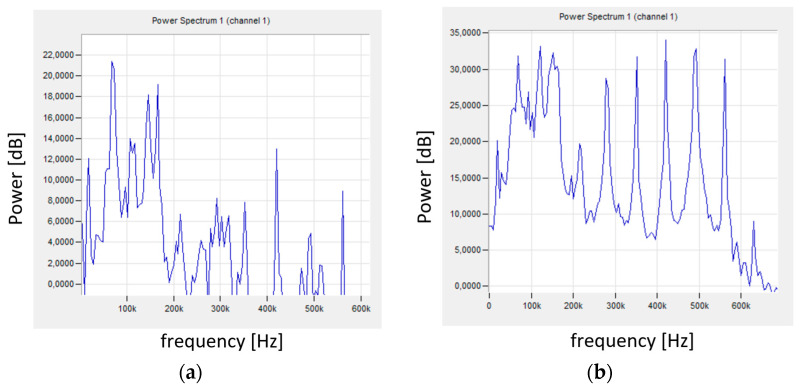
The AE signal spectrum for sample No. 4 (**a**) for the initial test phase—for stable machining conditions; (**b**) chip wrapping.

Figure 12a shows the signal spectrum in the initial phase of the test, while Figure 12b shows the signal spectrum in the later phase, where a ribbon chip was formed and stuck between the tool and the shaft. The signal spectrum shown in Figure 12a is similar to that obtained for stable turning conditions shown in Figure 9b. Chip wrapping resulted in the generation of a signal with changed characteristics (Figure 12b), where an increase in the power of the low-frequency signal was visible. Higher signal frequencies indicated increased machining instability. Additional chip friction on the surface of the shaft and the tool caused an increase in the signal energy. with frequencies mainly in the range of 20–165 kHz.

**Figure 13:**
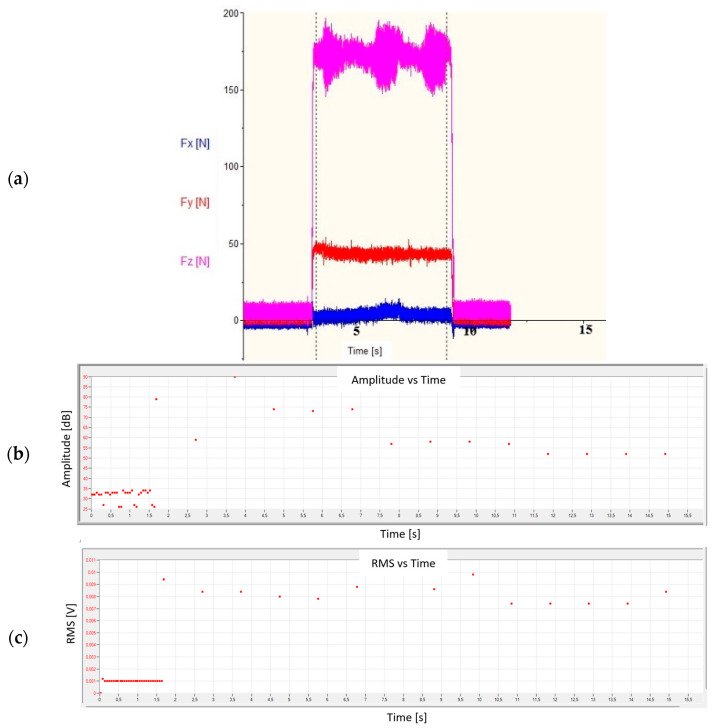
View of (**a**) forces and (**b,c**) the AE parameters during the turning process for unstable conditions—recorded for sample No. 9.

Figure 13 shows the recorded forces and the AE parameters—amplitudes and RMS for the following cutting parameters: Vc = 225 m/min, ap = 1.0 mm, and f = 0.236 mm/rev. When the machining conditions were not properly selected, the turning process was disturbed by uncontrolled chip formation in the cutting zone. This caused jumps in values for individual force components and the amplitude and RMS values of the AE signal.

**Figure 14:**
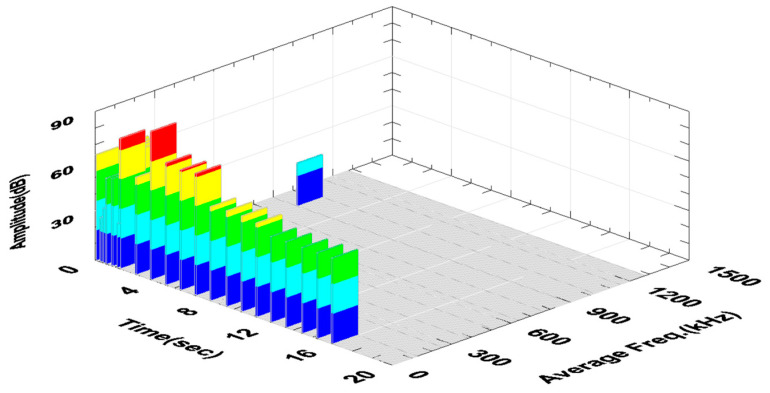
Change in the amplitude and the mean frequency of the AE signal as a function of time for sample No. 9, where unstable machining conditions were observed.

The fluctuations of the AE signal parameters recorded in the time interval from 4 to 10 s of the test may indicate these unstable machining conditions. This is particularly visible in the graph shown in Figure 14.

**Figure 15:**
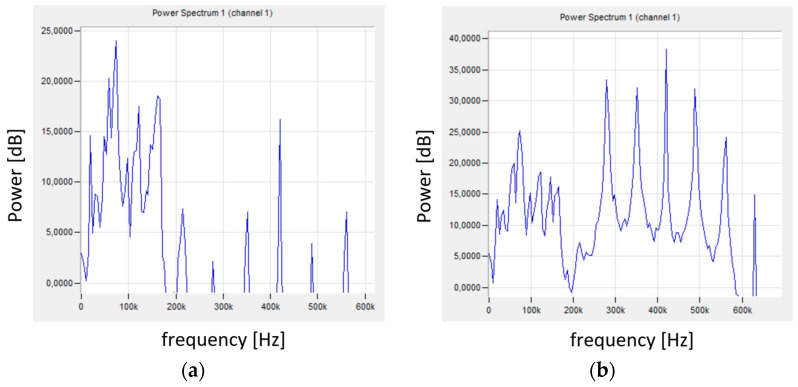
The AE signal spectrum for sample No. 9 for the initial test phase: (**a**) stable machining conditions; (**b**) unstable conditions.

Figure 15 shows the frequency spectra of the signals recorded during turning under stable (Figure 15a) and unstable (Figure 15b) machining conditions. The signals recorded for stable turning conditions for shafts machined with different parameters were characterized by a similar spectrum (similar frequency and signal power—Figure 9b, Figure 12a and Figure 15a).

The signal frequencies for stable and unstable conditions had similar values, but the signal power changed significantly. This applies to the high-frequency signal, above 200 kHz. Despite the changes in the signals with frequencies exceeding 200 kHz, their energy was so small that it was not visible on the graph (Figure 14) showing the change in amplitude. It also did not affect the average signal frequency.

Table 9 presents the selected parameters: amplitude, RMS, energy, and average frequency of the AE signal recorded during the tool contact with the workpiece. The signal generated in the initial phase of the test after switching on the spindle revolutions and automatic feed was not included in the analysis. The signals were analyzed only when the tool edge started cutting the material.

Stable machining conditions occur when a small scatter of results is recorded (small standard deviation). A similar approach to the analysis of research results was presented in the works of Shah et al. [20] and Tran et al. [21]. Among other things, they evaluated the RMS values of the acoustic emission signal, along with an in-depth analysis of its standard deviation. The smallest deviations were recorded for samples Nos. 1, 2, and 10, i.e., those where the feed was the smallest. This confirms the theory commonly known from the literature and the results of surface roughness tests. The largest scatter of the signal amplitude was recorded for sample No. 3, but it was not noticeable in the RMS and signal energy. The highest RMS and energy values were observed for sample No. 4. For the same sample, these parameters were also characterized by the largest standard deviation, which may indicate deterioration of the turning conditions. The results of the roughness tests confirmed the highest Ra value. With the machining parameters set for sample No. 4, a ribbon chip was formed, which also had an impact on the deterioration of the machined surface quality. Similarly, considering the standard deviation of the average signal frequency allows for a preliminary determination of the stability of the conducted process. Difficulties in unequivocally determining the effect of recorded AE signal parameters on the quality of the machined surface may result from a large range of cutting parameters used. Therefore, in subsequent studies, cutting parameters for finishing will be used, and their change will be in smaller ranges.

**Figure 16:**
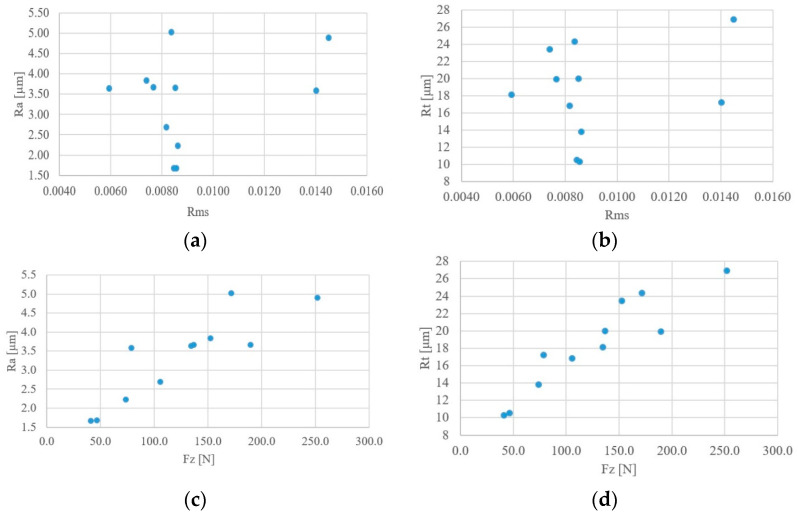
Cumulative results of the dependence of the AE RMS changes (**a**,**b**) and the Fz force (**c**,**d**) on the surface roughness parameters: Ra and Rt.

To sum up, Figure 16 presents the dependence of the influence of selected parameters, RMS of the AE signal and the Fz force, on the change of surface roughness parameters Ra and Rt.

While it is impossible to unambiguously determine the dependence of changes in the surface roughness parameters on the RMS of the recorded AE signal, it is visible that with increasing Fz force, the Ra and Rt parameters increase.

The previously mentioned planned future studies on smaller changes of machining parameters will complement the existing database and allow for more precise monitoring of the turning process using the acoustic emission method.

## 4. Conclusions

The turning process tests carried out on a conventional lathe show only the basic impact of input factors on the process of the analyzed machining and its impact on selected parameters of the geometric structure of the surface. A small change in the selected cutting parameters results in changes in the chip formation process and a change in the individual components of forces in the cutting zone. Manufacturers of cutting inserts provide recommended cutting parameters and their ranges during the insert’s operation; however, all factors related to the turning process impact the course and nature of the machining. The obtained results of measurements of the surface roughness parameters, the material ratio, and the recorded components of the cutting force, together with the analysis of the standard deviation, allow for determining stable machining conditions, ensuring the required quality of the part.

- Surface roughness parameters, especially Ra (1.67 μm), reached the lowest value for sample No. 2 machined with the following parameters: Vc = 300 m/min, ap = 0.5 mm, f = 0.078 mm/rev. The worst surface quality was obtained for samples Nos. 4 and 9 (Ra = 4.90 μm and 5.03 μm, respectively) turned with the feed rate f = 0.236 mm/rev, while the remaining parameters, Vc and ap, did not have such a significant effect on the surface roughness parameters. The remaining parameters of the surface roughness description, Rt, Rk, and Rvk, showed similar results. Only the analysis of the Rpk parameter showed different results compared to the analysis of the remaining parameters considered, which allows us to state that it has the lowest diagnostic value and should not be taken into account in future studies.
- The measurement of forces during turning indicated extreme conditions in terms of their stability. The lowest force Fz = 41 N was recorded for sample No. 2, the highest—for sample No. 4 (Fz = 251.8 N).
- Analysis of the dispersion of acoustic emission parameter values considered during the tests allowed for determining the stability of the turning process. For the most stable rolling conditions (sample No. 2), the smallest standard deviation of the recorded parameters was obtained: amplitude, RMS, energy, and average frequency. The most unstable machining conditions (sample No. 4) were determined in the same way—the highest standard deviation parameters were recorded: RMS and energy. In the case of such parameters as amplitude and average frequency, the results were ambiguous. The acoustic emission method allows for determining the best turning conditions, but in the case of determining the worst conditions, the results are ambiguous.

All the methods used in the tests, i.e., surface roughness measurement, testing of forces during turning, and acoustic emission, clearly indicated the best machining conditions, which means that they can be successfully used as tools for optimizing the selection of turning parameters of AW-7020 alloy shafts.

The results obtained under real machining conditions are significant from the perspective of creating a database for the turning process of the investigated alloy using conventional machines. The research may serve as a foundation for expanding the existing databases related to the machining of materials similar to AW-7020 under comparable conditions. The collected data can also be used as a basis for machine learning applications aimed at predicting machining conditions and optimizing the process.

## Figures and Tables

**Table 1:** The chemical composition of the tested EN AW-7020 alloy.

	Chemical Composition [wt. %]									
	Si	Fe	Cu	Mn	Mg	Cr	Zn	Ti	Zr	Al
Min	0	0	0	0.05	1.0	0.1	4.0	0	0.08	Balance
Max	0.35	0.40	0.20	0.5	1.4	0.35	5.0	0.1	0.2	
Real	0.17	0.11	0.04	0.18	1.19	0.12	4.65	0.03	0.13	Balance

**Table 2:** Research program on the influence of cutting parameters on the analyzed surface roughness parameters and material ratio.

Test No.	Coded Study Factors			Real Factors Studied (Independent Variables)		
	X_Vc_	X_ap_	X_f_	Vc [m/min]	ap [mm]	f [mm/rev]
1	−1	−1	−1	150	0.5	0.078
2	+1	−1	−1	300	0.5	0.078
3	−1	+1	−1	150	1.5	0.078
4	+1	+1	+1	300	1.5	0.236
5	−1	0	0	150	1.0	0.168
6	+1	0	0	300	1.0	0.168
7	0	−1	0	225	0.5	0.168
8	0	+1	0	225	1.5	0.168
9	0	0	+1	225	1.0	0.236
10	0	0	−1	225	1.0	0.078
11	0	0	0	225	1.0	0.168

**Table 3:** Results of the basic statistical analysis of the Ra parameter (highlighted in green—best, red—worst).

Hartley Plan Sample No.	Cutting Parameters (Independent Variables)				Ra [µm]			
	Vc [m/min]	ap [mm]	f [mm/rev]	Mean	Std. Dev.	Std. Error	Min	Max
1	150	0.5	0.078	1.68	0.13	0.07	1.55	1.80
2	300	0.5	0.078	1.67	0.02	0.01	1.66	1.69
3	150	1.5	0.078	2.69	0.35	0.20	2.34	3.04
4	300	1.5	0.236	4.90	0.10	0.06	4.82	5.01
5	150	1.0	0.168	3.84	0.08	0.05	3.75	3.89
6	300	1.0	0.168	3.64	0.02	0.01	3.62	3.66
7	225	0.5	0.168	3.59	0.01	0.01	3.58	3.60
8	225	1.5	0.168	3.67	0.11	0.06	3.56	3.78
9	225	1.0	0.236	5.03	0.16	0.09	4.84	5.13
10	225	1.0	0.078	2.23	0.17	0.10	2.1	2.42
11	225	1.0	0.168	3.66	0.08	0.04	3.58	3.73

**Table 4:** Results of the basic statistical analysis of the Rt parameter (highlighted in green—best, red—worst).

Hartley Plan Sample No.	Cutting Parameters (Independent Variables)				Rt [µm]			
	Vc [m/min]	ap [mm]	f [mm/rev]	Mean	Std. Dev.	Std. Error	Min	Max
1	150	0.5	0.078	10.51	1.58	0.91	9.26	12.28
2	300	0.5	0.078	10.30	0.58	0.33	9,92	10.97
3	150	1.5	0.078	16.85	2.49	1.44	14.02	18,73
4	300	1.5	0.236	26.92	1.99	1.15	24.98	28.96
5	150	1.0	0.168	23.43	0.77	0.45	22.58	24.08
6	300	1.0	0.168	18.13	0.98	0.56	17.17	19.12
7	225	0.5	0.168	17.24	0.47	0.27	16.75	17.69
8	225	1.5	0.168	19.91	0.25	0.14	19.67	20.16
9	225	1.0	0.236	24.34	0.45	0.26	24.04	24.93
10	225	1.0	0.078	13.81	2.19	1.26	11.93	16.21
11	225	1.0	0.168	20.01	2.55	1.47	18.26	22.94

**Table 5:** Results of the basic statistical analysis of the Rk parameter (highlighted in green—best, red—worst).

Hartley Plan Sample No.	Cutting Parameters (Independent Variables)				Rk [µm]			
	Vc [m/min]	ap [mm]	f [mm/rev]	Mean	Std. Dev.	Std. Error	Min	Max
1	150	0.5	0.078	5.58	0.49	0.29	5.26	6.15
2	300	0.5	0.078	5.89	0.12	0.07	5.78	6.01
3	150	1.5	0.078	8.96	1.44	0.83	8.97	10.39
4	300	1.5	0.236	17.80	0.70	0.40	17.00	18.29
5	150	1.0	0.168	13.81	0.53	0.31	13.27	14.33
6	300	1.0	0.168	13.70	0.15	0.09	13.53	13.82
7	225	0.5	0.168	13.25	0.30	0.17	13.06	13.60
8	225	1.5	0.168	15.01	2.36	1.37	13.62	17.74
9	225	1.0	0.236	18.71	0.45	0.26	18.19	19.02
10	225	1.0	0.078	7.34	0.34	0.20	6.95	7.55
11	225	1.0	0.168	13.81	0.16	0.09	13.63	13.90

**Table 6:** Results of the basic statistical analysis of the Rvk parameter (highlighted in green—best, red—worst).

Hartley Plan Sample No.	Cutting Parameters (Independent Variables)				Rvk [µm]			
	Vc [m/min]	ap [mm]	f [mm/rev]	Mean	Std. Dev.	Std. Error	Min	Max
1	150	0.5	0.078	1.89	0.22	0.13	1.66	2.09
2	300	0.5	0.078	1.09	0.08	0.05	1.01	1.17
3	150	1.5	0.078	2.78	0.16	0.09	2.61	2.93
4	300	1.5	0.236	4.01	0.52	0.30	3.59	4.6
5	150	1.0	0.168	3.17	0.53	0.31	2.75	3.77
6	300	1.0	0.168	1.32	0.34	0.19	0.99	1.66
7	225	0.5	0.168	1.37	0.13	0.08	1.22	1.46
8	225	1.5	0.168	2.79	0.35	0.20	2.39	3.00
9	225	1.0	0.236	3.56	1.20	0.69	2.20	4.48
10	225	1.0	0.078	2.50	0.68	0.39	1.91	3.24
11	225	1.0	0.168	2.21	0.72	0.42	1.76	3.04

**Table 7:** Results of the basic statistical analysis of the Rpk parameter (highlighted in green—best, red—worst).

Hartley Plan Sample No.	Cutting Parameters (Independent Variables)				Rpk [µm]			
	Vc [m/min]	ap [mm]	f [mm/rev]	Mean	Std. Dev.	Std. Error	Min	Max
1	150	0.5	0.078	1.26	0.35	0.20	0.99	1.65
2	300	0.5	0.078	1.58	0.13	0.08	1.45	1.71
3	150	1.5	0.078	2.91	0.68	0.39	2.13	3.33
4	300	1.5	0.236	2.09	0.29	0.17	1.76	2.29
5	150	1.0	0.168	2.92	0.84	0.48	2.30	3.87
6	300	1.0	0.168	2.00	0.24	0.14	1.72	2.17
7	225	0.5	0.168	1.27	0.08	0.04	1.18	1.32
8	225	1.5	0.168	1.86	0.27	0.15	1.70	2.17
9	225	1.0	0.236	2.18	0.17	0.10	2.01	2.35
10	225	1.0	0.078	1.93	0.48	0.28	1.48	2.44
11	225	1.0	0.168	2.06	1.20	0.69	1.26	3.44

**Table 8:** Average, minimum, and maximum values of forces during the turning process (highlighted in green—best, red—worst).

Hartley Plan Sample No.	Fx [N]	Min	Fy [N]	Min	Fz [N]	Min
		Max		Max		Max
1	−4.4	−46.4	18.3	10.1	46.7	31.9
		1.7		34.9		93.3
2	−2.2	−8.3	11.9	8.1	41.0	29.1
		3.7		16.2		53.9
3	2.5	−5.9	43.0	30.8	105.7	89.1
		9.8		58.1		123.8
4	14.2	−59.3	50.2	33.2	251.8	150.4
		175.4		168.1		604.2
5	8.9	−2.1	56.4	34.8	152.4	112.7
		17.8		84.2		187.2
6	3.8	−3.4	31.3	23.2	134.6	106.4
		20.0		37.4		162.8
7	−2.5	−32.8	16.9	3.9	78.9	54.4
		38.7		31.5		141.9
8	8.2	0.5	52.1	41.0	189.7	154.5
		16.2		62.5		219.7
9	3.6	−5.0	43.6	34.4	171.8	145.5
		14.2		54.0		196.5
10	−0.3	−6.6	26.5	19.8	73.8	60.6
		5.7		32.5		87.2
11	3.6	−4.4	34.8	27.0	136.8	113.8
		9.8		41.1		157.2

**Table 9:** Average, minimum, and maximum values of the selected AE parameters (highlighted in green—best, red—worst).

Hartley Plan Sample No.	Amplitude [dB]		RMS [mV]		Energy [eu]		Avg. Frequency [kHz]	
	Avg.	Std. Dev	Avg.	Std. Dev.	Avg.	Std. Dev.	Avg.	Std. Dev.
1	54.80	6.76	8.46	0.39	8277	560	14.7	3.1
2	53.54	0.52	8.55	0.25	8877	248	13.5	2.9
3	70.35	21.13	8.18	1.59	5941	2491	15.2	12.5
4	66.20	10.75	14.50	6.96	13,270	5087	20.1	10.9
5	82.71	6.34	7.40	2.30	6044	2016	10.7	6.6
6	71.50	15.55	5.93	2.78	6380	2522	20.2	14.5
7	76.90	14.92	14.02	4.22	11,576	2307	17.3	9.4
8	67.08	14.60	7.66	0.55	7777	628	7.7	9.2
9	61.43	11.70	8.37	1.02	8903	1546	15.1	9.1
10	53.38	1.02	8.62	0.43	8687	483	16.4	3.4
11	70.83	15.75	8.52	1.55	7770	1956	10.5	8.7

## Data Availability

The original contributions presented in this study are included in the article. Further inquiries can be directed to the corresponding author.
